# Guideline interval: A new time interval in the diagnostic pathway for symptomatic cancer

**DOI:** 10.1016/j.canep.2021.101969

**Published:** 2021-08

**Authors:** Sarah Price, Gary A. Abel, Willie Hamilton

**Affiliations:** a College House, University of Exeter Medical School, St Luke’s Campus, Heavitree Road, Exeter, EX1 2LU, UK; b Smeall Building, University of Exeter, St Luke’s Campus, Heavitree Road, Exeter, EX1 2LU, UK

**Keywords:** Databases and data mining, Electronic health records, Modelling healthcare services, Diagnostic interval

## Abstract

**Background:**

A standard measure of the cancer diagnostic pathway, diagnostic interval, is the time from “first presentation of cancer” to diagnosis. Cancer presentation may be unclear in patients with multimorbidity or non-specific symptoms, signs or test results (“features”). We propose an alternative, guideline interval, with a more certain start date; namely, when the patient first meets suspected-cancer criteria for investigation or referral.

**Methods:**

This retrospective cohort study used Clinical Practice Research Datalink (CPRD) and English cancer registry data. Participants, aged ≥55 years, had diagnostic codes for oesophagogastric cancers in 1/1/12–31/12/17. Features of oesophagogastric cancer in the year before diagnosis were identified from CPRD codes for dysphagia, haematemesis, upper-abdominal mass or pain, low haemoglobin, reflux, dyspepsia, nausea, vomiting, weight loss or thrombocytosis. Diagnostic interval was the time from first feature to diagnosis; guidance interval, the time from first meeting criteria in NICE suspected-cancer guidance to diagnosis. Multimorbidity burden was quantified using Adjusted Clinical Groups®. Accelerated failure-time models explored associations between multimorbidity burden and length of both diagnostic and guideline interval.

**Results:**

There were 3,793 eligible participants (69.0 % male), mean age 74.1 years (SD 10.5). 3,097 (81.7 %) presented with ≥1 feature in the year before diagnosis, and 1,990 (52.5 %) met NICE suspected-cancer criteria. The median for both intervals was 11 days in healthy users, and rose with increasing morbidity burden. At very high multimorbidity burden, diagnostic interval was 5.47 (95%CI 3.25–9.20) times longer and guideline interval was 3.91 (2.63–5.80) times longer than for healthy users.

**Conclusions:**

Guideline interval is proposed as a new measure of the cancer diagnostic pathway. It has a more certain start date than diagnostic interval, and is lengthened less than diagnostic interval in people with a very high multimorbidity burden. Guideline interval has potential for assessing the implementation of suspected-cancer policies.

## Highlights

• We describe a new metric to describe the cancer diagnostic pathway: guideline interval.
• Guideline interval increases less with multimorbidity compared with diagnostic interval.
• We illustrate guideline interval using UK NICE suspected-cancer referral guidance.
• Guideline interval is readily modifiable to local policies or updates.
• Guideline interval is readily adaptable to local suspected-cancer policies.

## 1 Introduction

Timely diagnosis of cancer to improve patient outcomes is a UK government priority. Policies focus on increasing the proportion of cancers diagnosed early (i.e. at stage 1 or 2), from a half to three-quarters by 2028 [1]. Monitoring policy effectiveness requires robust measures of key time points along the diagnostic pathway. One frequently used metric is the diagnostic interval, defined as the time between the first presentation of cancer to healthcare and diagnosis [2].

The “first presentation of cancer” can be difficult to discern, particularly from health care records. The Aarhus statement defines it as “the time point at which, given the presenting signs, symptoms, history and other risk factors, it would be at least possible for the clinician seeing the patient to have started investigation or referral for possible important pathology, including cancer” [2]. The standard approach is to regard the first presentation as being the first recorded feature of possible cancer (i.e. symptom, sign or abnormal test result) in the year before diagnosis [[3], [4], [5]]. This approach is problematic because presenting features have varying positive predictive values (PPV) for the underlying cancer, and cancers may present with a narrow or broad selection of features [6]. High-risk features include a breast lump, with a PPV of over 8% for breast cancer in women aged 50–59 [7]. In such cases, it is likely that the breast lump is the first presenting feature. Other features are low-risk and may also be non-site-specific. An example is abdominal pain, which has a PPV of around 2.4 % for any cancer, made up from colorectal (1.5 %), oesophagogastric (0.3 %), pancreatic (0.3 %) or bladder/renal (0.2 %), plus a number of rarer cancers [8]. It is less clear whether such features meet the above-described criteria for first possible presentation, as the clinician will not be sure enough of the possible cancer site to initiate the appropriate investigation or referral. Furthermore, most features of possible cancer have other, non-cancer, causes. For example, lung cancer and chronic obstructive pulmonary disease both present with cough and dyspnoea [9]. Consistent with this, 27%–48% of the apparent first symptoms of possible lung cancer may not be caused by the malignancy at all and may represent a constant background level of these symptoms caused by non-cancerous conditions [10].

Arguably, the uncertainty in the starting point for diagnostic interval is greater for first presenting symptoms that are low-risk and nonspecific. Therefore, the diagnostic interval is more likely to be uncertain for cancers with broad symptom signatures, and in patients with co-morbidities [6,11]. This leads to difficulties in interpretation of diagnostic interval data. For example, does the longer diagnostic interval in patients with conditions that share symptoms with colorectal cancer represent genuinely delayed diagnosis, or is it an artefact [12]?

In this paper, our primary objective is to derive a new metric to describe the diagnostic pathway, the guideline interval for diagnosis. We propose that the guideline interval will be less uncertain than diagnostic interval because of its more objective start date. Furthermore, it aligns with a key early-diagnosis policy, the National Institute for Health and Care Excellence suspected-cancer recognition and referral guidance (NG12) [8]. A second objective is to compare the characteristics of the guideline and diagnostic intervals, by examining data distributions and exploring their associations with patient characteristics, such as morbidity burden. We use oesophagogastric cancer as an example, as it presents with a broad range of features, including high-risk dysphagia and haematemesis, and low-risk features, dyspepsia, back pain, and abdominal pain, which are also non-site-specific being shared with other benign and self-limiting conditions [13]. Oesophagogastric cancers tend to be diagnosed at an advanced stage and, compared with rectal cancer, patients are more likely to require three or more consultations in primary care before they are referred for investigation [14].

## 2 Methods

### 2.1 Dataset

This primary care study used UK Clinical Practice Research Datalink (CPRD) GOLD data with English cancer registry linkage (set 15). The dataset contains the coded portion of anonymised electronic records of over 11.3 million patients from 674 UK general practices. Information on clinical history, diagnoses, prescriptions, test results, investigations, and referrals is collected from participating practices adhering to stringent data quality and validation checks [15].

### 2.2 Inclusion and exclusion criteria

Lists of diagnostic codes for oesophagogastric cancer (ICD10 C15 or C16, and equivalent Read codes) were collated [16], and used to identify cases diagnosed between 1 January 2012 and 31 December 2017. The diagnosis date was taken as the earliest recorded diagnostic code in linked registry data, or the earliest CPRD diagnostic code for cases without linked data. Cases were restricted to those aged ≥55 years on their incident oesophagogastric cancer diagnostic code, this being the age threshold in the NICE suspected-cancer referral guidance [8,17,18]. Cases had to have attended the general practice at least once in the year before diagnosis, and to have been registered continuously there for that year or longer.

### 2.3 Outcome variables

The two main outcome variables were the conventional metric *diagnostic interval* [2] and the new measure *guideline interval*. Both were calculated as the number of days between an “index date” and the diagnosis date, constrained to a maximum of one year before diagnosis. The definitions of index date were:

• For *diagnostic interval*: the date of the first CPRD record of any feature of possible oesophagogastric cancer to primary care (Table 1).

**Table 1:** Features of oesophagogastric cancer, and the criteria for determining their index date in diagnostic interval and guideline interval.

Feature of undiagnosed oesophageal cancer	Diagnostic interval criteria	Guideline interval criteria
Dysphagia	Alone	Alone
Haematemesis	Alone	Alone
Upper abdominal mass	Alone	Alone
Low haemoglobin	Alone	Concurrent with abdominal pain
Reflux	Alone	Concurrent with weight loss, raised platelet count, nausea or vomiting
Upper abdominal pain	Alone	Concurrent with weight loss, raised platelet count, nausea or vomiting
Dyspepsia	Alone	Only treatment-resistant or concurrent with weight loss, raised platelet count, nausea or vomiting
Nausea and/or vomiting	Alone	Concurrent with raised platelet count, weight loss, reflux, dyspepsia or upper abdominal pain
Weight loss	Alone	Concurrent with raised platelet count, upper abdominal pain, reflux, dyspepsia, nausea or vomiting
Raised platelet count	Alone	Concurrent with nausea, vomiting, weight loss, reflux, dyspepsia, or upper abdominal pain
• For *guideline interval*: the date that the CPRD record indicates that the participant first met any criterion for clinical action (Table 1) in NICE referral guidelines (NG12) for suspected oesophagogastric cancer [8,19].

### 2.4 Identification of index date

#### 2.4.1 For diagnostic interval

Presentation of possible oesophagogastric cancer was defined as a recorded attendance for any symptom, sign or abnormal blood test result in NICE guidance for suspected oesophagogastric cancer (Table 1) [8,19]. Read code lists for each feature were collated, and occurrences of these in the year before diagnosis were identified [16]. The variable for low haemoglobin compared each participant’s haemoglobin result with their local laboratory’s reference range.

#### 2.4.2 For guideline interval

All coded records of upper abdominal mass were assumed to be “consistent with stomach cancer”. Participants were deemed to have treatment-resistant dyspepsia if they had a prescription code for a proton pump inhibitor at least 8 weeks before their dyspepsia code. Many of the NICE criteria consist of multiple concurrent clinical features. “Concurrent” was defined as occurring within a 1-month window, with the index date assigned to be the date that the last feature occurred (i.e. when the criterion was fully met).

### 2.5 Data visualisation

The distributions of raw and log-transformed diagnostic and guideline intervals were examined. Log transformation was used because of its suitability for very right-skewed data. Furthermore, log-transformation implies multiplicative differences are being considered (rather than additive ones). For example, after log-transforming the data the difference between 1 day and 10 days is the same as the difference between 10 days and 100 days (i.e. a 10-fold increase in interval).

### 2.6 Exposure variables

Participant-level multimorbidity burden was estimated from the Johns Hopkins Adjusted Clinical Groups (ACG)® system (version 11.2.1) resource utilisation band, which has five categories: 0, no diagnoses; 1, healthy users; 2, low-level; 3, moderate-level; 4, high-level; or 5, very high resource utilisation. Patients in each band share similar multimorbidity burden, in terms of their expected use of healthcare resources. The final categorisation is achieved by ACG® software, following searches of CPRD records in the year before the index date for diagnostic and therapeutic codes. Searches are extended to 5 years before the index date for long-term conditions listed in the Quality and Outcomes Framework (QOF) register [20]: asthma, atrial fibrillation, cancer, coronary heart disease, chronic kidney disease, dementia, diabetes, epilepsy, heart failure, hypertension, learning disabilities, schizophrenia and other psychoses, stroke, hypothyroidism, and cardiovascular disease. ACG® code lists are available from the authors.

We identified which participants had pre-existing condition(s) in the year before the index date that might provide a plausible diagnostic alternative for features of oesophagogastric cancer; namely:

• *Dysphagia*: Parkinson’s disease, oesophageal stricture, stroke (also a QOF condition)
• *Weight loss*, *Anaemia*: Inflammatory bowel disease, chronic kidney disease (also a QOF condition)
• *Nausea*, *Vomiting*, *Upper abdominal pain*: Hernia, pancreatitis, ulcer, gastritis, oesophagitis, irritable bowel syndrome
• *Haematemesis*: Anticoagulant medications (note the need for anticoagulation was treated as a “condition”)
• *Dyspepsia/reflux*: Oesophagitis, gastritis

Sex and age were identified from CPRD variables, assigning a birthday of 1st July, as the CPRD only releases year of birth to protect anonymity. An indicator variable for cancer site (stomach or oesophagus) was identified from the CPRD or Registry diagnostic code. In line with standard practice, we interpreted the absence of a Read code as non-occurrence of that clinical event [15].

### 2.7 Analyses

For both diagnostic and guideline intervals, associations with morbidity burden and alternative explanations were explored using accelerated failure time models. These models were chosen over the standard Cox model, as the proportional hazards assumption was violated for analysis of diagnostic but not guideline interval (results not shown). Additionally, we were interested in whether there were differences in the underlying functional form for the two different intervals. Finally, we opted for the accelerated failure time models, as the coefficients are readily interpretable. Univariable and multivariable analyses are reported as time ratios (TR), where a time ratio >1 (or <1) reports the factor by which a covariate prolongs (or shortens) the time to diagnosis (i.e. the interval). Models adjusted for cancer site (stomach or oesophagus), age, sex, and used the clustered sandwich estimator to relax the requirement for observations within practices to be independent. Analyses were run using Weibull, log-normal, log-logistic and exponential distributions, and the Akaike information criterion was used to select the best parameterisation of the data.

## 3 Results

### 3.1 Sample characteristics

The CPRD provided 3,806 participants aged ≥55 years diagnosed with oesophagogastric cancer. After excluding 13 non-attenders in primary care, 3,793 participants (69.0 % male; 65.2 % oesophageal cancer) were included in the analyses (Table 2). Participant characteristics were similar for both sexes. Mean (standard deviation) age at diagnosis was 74.1 (10.5) years. The majority of participants fell into the moderate (2,403/3,793, 63.4 %) or high (839/3,793, 22.1 %) morbidity burden bands. Having diagnostic alternatives for cancer features was uncommon (5.7 %).

**Table 2:** Participant characteristics.

		Male (n = 2,617)	Female (n = 1,176)	Total (N = 3,793)
Site	Oesophagus, n (%)	1,713 (65.5)	759 (64.5)	2,472 (65.2)
	Stomach, n (%)	904 (34.5)	417 (35.4)	1,321 (34.8)
Age, mean (SD)		73.0 (9.7)	76.5 (10.3)	74.1 (10.1)
Presented with a feature of possible cancer, n (%)		2,126 (81.2)	971 (82.6)	3,097 (81.7)
Met a NICE criterion		1,368 (52.3)	622 (52.9)	1,990 (52.5)
Had a diagnostic alternative, n (%)		137 (5.2)	79 (6.7)	216 (5.7)
Morbidity burden	No or only non-valid diagnoses	49 (1.9)	22 (1.9)	71 (1.9)
	Healthy user	60 (2.3)	24 (2.0)	84 (2.2)
	Low	132 (5.0)	45 (3.8)	177 (4.7)
	Moderate	1,664 (63.6)	739 (62.8)	2,403 (63.4)
	High	567 (21.7)	272 (23.1)	839 (22.1)
	Very high	145 (5.5)	74 (6.3)	219 (5.8)

### 3.2 Diagnostic and guideline intervals

The majority of participants (3,097/3,793, 81.7 %) had at least one coded feature of possible cancer in the year before diagnosis, allowing estimation of diagnostic interval. Fewer (1,990/3,793, 52.5 %) met a NICE criterion allowing estimation of their guideline interval.

#### 3.2.1 By morbidity burden

Median (interquartile range) guideline and diagnostic intervals both increased with increasing morbidity burden (Table 3), from a similar base in healthy users (11 (7–24) days and 11 (7–27) days, respectively, n = 47). Increases with morbidity burden were more marked for diagnostic interval than for guideline interval. By definition, the diagnostic interval has to be equal to or longer than the guideline interval, and diagnostic intervals of up to a year are seen for all values of guideline interval. Overall, the median diagnostic interval was 22 days longer than the median guideline interval, with increasing differences at increasing level of multimorbidity burden (Table 3).

**Table 3:** Diagnostic and guideline intervals by morbidity burden.

Morbidity burden	n	Diagnostic interval (days) at centile:				Guideline interval (days) at centile			
		50^th^	25^th^	75^th^	90^th^	50^th^	25^th^	75^th^	90^th^
No diagnoses	19	46	22	109	173	22	14	38	123
Healthy user	47	11	7	27	63	11	7	24	49
Low	93	30	14	69	196	22	13	56	97
Moderate	1,278	54	23	156	275	36	16	78	166
High	445	85	35	232	320	46	22	101	207
Very high	108	138	58.5	280	343	51.5	19.5	138	261
Overall	1,990	58.5	24	174	291.5	36.5	16	81	180

#### 3.2.2 Distributions

**Fig. 1:**
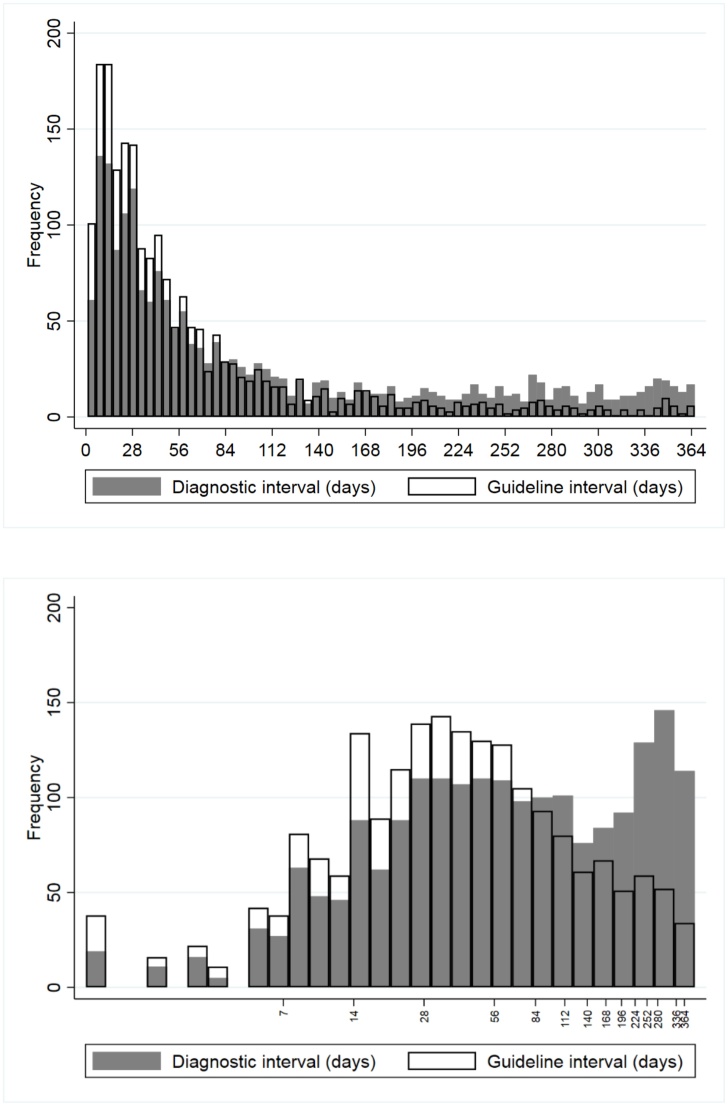
Overlaid histograms of guideline (clear bars) and diagnostic (grey bars) interval, constrained to 1 year before diagnosis. Upper panel: raw data; lower panel: log-transformed data.

Both diagnostic and guideline interval had positively skewed distributions (Fig. 1, upper panel). After log-transformation, diagnostic interval tended towards a bimodal distribution, whereas guideline interval had a near-symmetrical distribution (Fig. 1, lower panel). The nadir between the two modes in the diagnostic interval distribution occurs between 4 and 5 months.

#### 3.2.3 Diagnostic and guideline intervals: associations with morbidity burden and alternative explanations

Guideline and diagnostic interval data were best parameterised by log normal and Weibull distributions, respectively. For both intervals, there was strong evidence of a positive association with increasing morbidity burden, adjusting for cancer site, age, and sex (Table 4). Compared with healthy users, even a low morbidity burden was associated with a more than doubling of guideline interval (time ratio 2.11, 95%CI 1.48–3.00, p < 0.0001) and diagnostic interval (2.21, 1.24–3.97, p = 0.007). The association was greater for diagnostic interval than for guideline interval. For very high morbidity burden, diagnostic intervals were 5.47 (3.25–9.20, p < 0.0001) longer and guideline intervals were 3.91 (2.63–5.80, p < 0.0001) longer than for healthy users.

**Table 4:** Accelerated failure time models for guideline and diagnostic intervals. Associations with diagnostic alternatives and resource utilisation band, adjusted for age, sex and cancer site (n = 1,990).

Explanatory variable		Guideline interval				Diagnostic interval			
		Unadjusted		Adjusted		Unadjusted		Adjusted	
		TR (95% CI)	p	TR (95% CI)	p	TR (95% CI)	p	TR (95% CI)	p
Cancer site (ref category: stomach)		0.81 (0.71 to 0.92)	0.002	0.80 (0.70 to 0.91)	0.001	0.78 (0.71 to 0.86)	<0.0001	0.81 (0.74 to 0.88)	<0.0001
Sex (ref category: male)		1.09 (0.97–1.23)	0.136	1.12 (1.00–1.26)	0.051	1.03 (0.93–1.14)	0.533	1.02 (0.93–1.13)	0.665
Age		1.00 (0.99–1.01)	0.887	0.99 (0.99–1.00)	0.020	1.01 (1.01–1.02)	<0.0001	1.00 (1.00–1.01)	0.108
Diagnostic alternative		0.93 (0.77–1.11)	0.420	0.85 (0.71–1.02)	0.077	1.23 (1.08–1.40)	0.002	1.10 (0.97–1.24)	0.152
Morbidity burden (ref category: healthy user)	No diagnoses	1.71 (0.96–3.04)	0.069	1.57 (0.88–2.82)	0.134	2.34 (1.17–4.27)	0.015	2.35 (1.21–4.56)	0.011
	Low	2.13 (1.50–3.02)	<0.001	2.11 (1.48–3.00)	<0.0001	2.28 (1.28–4.04)	0.005	2.21 (1.24–3.97)	0.007
	Moderate	2.84 (2.10–3.84)	<0.0001	2.88 (2.12–3.90)	<0.0001	3.55 (2.16–5.84)	<0.0001	3.35 (2.02–5.55)	<0.0001
	High	3.50 (2.53–4.81)	<0.0001	3.66 (2.65–5.06)	<0.0001	4.72 (2.86–7.79)	<0.0001	4.37 (2.62–7.26)	<0.0001
	Very high	3.58 (2.42–5.30)	<0.0001	3.91 (2.63–5.80)	<0.0001	5.93 (3.57–9.85)	<0.0001	5.47 (3.25–9.20)	<0.0001

There was no evidence that having alternative explanations for cancer features was associated with either interval.

## 4 Discussion

### 4.1 Summary

We describe a new measure, guideline interval, as a potential improvement in describing time to cancer diagnosis following symptomatic presentation. This measure compares favourably with the standard metric diagnostic interval in the following ways. First, median (interquartile range) guideline interval (11, 7–24 days, n = 47) and diagnostic interval (11, 7–27 days, n = 47) were similar in healthy users. While neither interval was associated with alternative explanations for cancer, the positive association with increasing morbidity burden was greater for diagnostic interval than for guideline interval. Furthermore, log-transformed guideline interval has a unimodal distribution, whereas log-transformed diagnostic interval has a bimodal distribution. This latter probably reflects the contribution of a constant background level of nonspecific features caused by conditions unrelated to the cancer. Second, guideline interval is highly suited to assessing the implementation and impact of NICE suspected-cancer referral guidance NG12 in England, as it aligns with the recommendations therein. Third, the measure can be readily adapted in response to updates to NG12, or to assess performance against standards of care in other countries, by modifying the clinical criteria to local policy.

Conversely, guideline interval has some limitations. It is more complicated to compute than diagnostic interval, particularly for criteria that consist of multiple features. It can only be estimated for people matching the criteria for cancer testing, which in this example was only just over half of patients, whereas the diagnostic interval can be calculated for all patients where symptoms of possible cancer are recorded. Whilst this may be a drawback for epidemiological studies, it is less relevant if the guideline interval is used as a marker of clinical performance against national policy guidance.

### 4.2 Research implications and recommendations

Patients meeting criteria for clinical action in NICE NG12 (or whichever policy under study) will always be a subset of all symptomatic patients. This limitation means that studies using guideline interval will have reduced power and generalisability compared with studies of diagnostic interval. Therefore, we outline some research recommendations that might reduce the uncertainty of the index date for diagnostic interval.

All previous studies seeking to identify cancer symptoms in primary care records have used a cut-off date, typically 1 year, beyond which attribution of symptoms to the cancer is deemed unreliable. This study suggests that symptom searches should be constrained to a period shorter than 1 year, as guided by the distribution of log diagnostic interval for each cancer. In the case of the cancers studied here, 5 months might represent a suitable cut-off. Further research using other cancer sites should examine this. It is also important to estimate the symptom lead time for all cancers [21]. This is defined as the time between symptoms *attributable* to cancer and eventual clinical diagnosis, and has only been done for lung (4.7–6.0 months) and colon (4.1–5.0 months) to date [10].

Our study suggests that time to diagnosis increases with multimorbidity burden, whether measured by guideline interval or the standard diagnostic interval. For diagnostic interval, it is not clear whether these longer diagnostic intervals represent diagnostic delay, or are an artefact associated with background presentation of features from non-cancer causes. For guideline interval, it is easier to identify if investigation is delayed with increasing multimorbidity burden [11]. Further research on decision-making around cancer testing in patients with multimorbidity, focusing on cancers presenting with a broad signature of nonspecific symptoms is recommended [6,11].

### 4.3 Strengths and limitations

A considerable strength is the study’s primary-care setting, where about 85 % of patients make their first presentation with possible cancer [22,23]. The data sources are further strengths: the CPRD is the largest primary-care database worldwide and is recognised for its high-quality data [15], and NCRAS provides complete coverage of all people diagnosed with cancer in England [24]. The current concordance rate between CPRD and NCRAS information is 83·3% [25], providing reassurance that the diagnosis type and date for participants without unlinked data introduces minimal error or bias.

We used robust methods to collate comprehensive code lists for features of undiagnosed cancer [16]. Nevertheless, we have to assume that either the GPs have sought, or that patients have reported, information about the cancer features of interest to our study, and that the GPs have coded this information rather than noting it an irretrievable part of the notes [15]. Inevitably there will have been some data loss. Our finding that 82 % of participants had coded features of cancer is consistent with evidence that coded CPRD data identifies 80 % of visible haematuria or jaundice events, and 60–70 % of abdominal pain in patients with pancreatic or bladder cancers [26]. A further strength is the use of the resource utilisation band (ACG® Johns Hopkins). This is a strong predictor of health-care use in the primary care setting, with greater validity than the Charlson Index and disease counts [27].

## 5 Conclusion

We report on a new, readily adaptable measure of the timeliness of cancer diagnosis – guideline interval – that is more robust to existing multimorbidity than the current standard, diagnostic interval. It is particularly suited for use in studies of outcomes compared with recommended standards of care or waiting times.

## Funding

The dataset had been used for a separate study on time to diagnosis for many cancers in a study funded by 10.13039/501100000289Cancer Research UK (Grant Reference Number 21550). This research was also part-funded by the National Institute for Health Research (NIHR) Policy Research Programme, Policy Research Unit in Cancer Awareness, Screening and Early Diagnosis (grant number PRU-1217-21601). This research is also linked to the CanTest Collaborative, which is funded by CRUK (grant reference number: C8640/A23385), of which Willie Hamilton is Co-Director and Gary Abel is a co-investigator. The views expressed are those of the author(s) and not necessarily those of the NIHR or the Department of Health and Social Care.

## CRediT authorship contribution statement

**Sarah Price:** Conceptualization, Methodology, Software, Formal analysis, Investigation, Data curation, Writing - original draft, Writing - review & editing. **Gary A. Abel:** Writing - review & editing, Supervision. **Willie Hamilton:** Conceptualization, Funding acquisition, Supervision, Writing - review & editing.

## Declaration of Competing Interest

The authors report no declarations of interest.
